# Supplementary material for: Intercomparison of atmospheric trace gas dispersion models: Barnett Shale case study
Source: Atmos Chem Phys. Author manuscript; Available in PMC 2019 Jul 2. (PMC6605086; doi:10.5194/acp-19-2561-2019)
Supplement: Sup1 [file NIHMS1528455-supplement-Sup1.pdf]

Supplement of Atmos. Chem. Phys., 19, 2561–2576, 2019
https://doi.org/10.5194/acp-19-2561-2019-supplement
© Author(s) 2019. This work is distributed under
the Creative Commons Attribution 4.0 License.

Atmospheric Chemistry and Physics
Open Access

Supplement of

Intercomparison of atmospheric trace gas dispersion models: Barnett Shale case study

Anna Karion et al.

Correspondence to: Anna Karion (anna.karion@nist.gov)

The copyright of individual parts of the supplement might differ from the CC BY 4.0 License.

Supplementary Material

S1 Evaluation of WRF-FDDA wind and PBL depth using Lidar and aircraft observations

To evaluate the performance of the WRF-FDDA model runs that we use in the dispersion calculation, we compare with observations from the field. Two sources of observations are used in this comparison: NOAA/ESRL Chemical Sciences Division (CSD)'s High-Resolution Doppler Lidar (HRDL), and the aircraft observations. The HRDL retrieves horizontal wind speed and direction, vertical wind speed variance, and aerosol scattering as a function of altitude at one stationary location where the instrument was deployed in the field (Fig. 1) (Grund et al., 2001); more information about this deployment is in Karion et al. (2015). HRDL was not operational for the first three flights in October 2013 due to the US federal government shutdown on those days.

Wind speed and direction observations from the HRDL allow for comparison with the WRF-FDDA model at one location but continuous in time, including the time period that is used in the STILT or HYSPLIT model runs, which extends 12 h – 24 h back from the flight time. Both the HRDL winds and the model were averaged in height from the ground to the top of the PBL as determined in WRF. Airborne measurements of the wind speed and direction provided an additional evaluation metric, with information about the wind across the entire field but only during the time of the flight. Comparisons indicate that the WRF-FDDA model, on most flight days, predicts average wind speed and direction quite well, with wind speed errors up to 30% (1.8 m s^{-1}) at most, with a mean high bias of 11% (0.7 m s^{-1}) over all the days relative to the aircraft and a low bias of 3% (0.1 m s^{-1}) relative to HRDL, with significant variability over the times of the averages (Fig. S1, S3, Table S1).

The WRF-FDDA PBL depth was evaluated against HRDL observations for the 12 hours prior to each flight, and against the PBL depth used for the MBE, from airborne observations (Fig. S2, S3, and Table S2). PBL depth was provided by NOAA/ESRL's Chemical Sciences Division as it was retrieved from the HRDL data using an algorithm developed for this purpose (Bonin et al., 2017). PBL depths used for the mass balance calculation in Karion et al. (2015), which were estimated from the aircraft vertical profiles during the flight, are compared with the WRF-FDDA PBL depths, averaged over the flight time.

Over all the flight days, the WRF-FDDA PBL depth showed a difference of 26 ± 26 % (mean ± standard deviation) relative to HRDL observations and 11 ± 14 % (mean ± standard deviation) relative to the aircraft observations, with the model biased towards low PBLs. Both wind and PBL comparisons indicate that relative errors in WRF-FDDA, on average, are not large. However, looking for specific days when the average winds or PBL are not faithful to the observations can serve as a possible metric to evaluate the derived emissions for given days. We can see that WRF wind speeds are often higher than the observations, especially those from the aircraft (a negative difference in Fig. S3), and on 20131016, 20131019, and 20131025, these differences are larger than other days (20%-30% vs. < 10% on other days). PBL depth on the other hand is often underestimated in the model (shown as a positive difference in Fig. S2).

S2 Model configuration details

Here we describe in detail the various configurations of the models that were run for this study and are listed in Table 1 in the main text.

S2.1 WRF

The primary transport model used in this analysis was the Weather Research and Forecasting (WRF) model, version 3.4, in Four Dimensional Data Assimilation (FDDA) mode. The WRF-FDDA configuration for the model physics includes the use of: 1) the Thompson microphysical processes, 2) the Grell 3-D ensemble scheme for cumulus parameterization on the coarse grid, 3) the Rapid Radiative Transfer Model (RRTM) for longwave atmospheric radiation, and the Dudhia scheme for shortwave atmospheric radiation, 4) the TKE-predicting Mellor-Yamada-Nakanishi-Niino (MYNN) Level 2.5 turbulent closure scheme for boundary layer turbulence parameterization, and 5) the 6-level RUC land surface model (LSM) for representation of the interaction between the land surface and the atmospheric surface layer. The WRF modeling system also has data assimilation (FDDA) capabilities to allow the meteorological observations to be continuously assimilated into the model. The WRF-FDDA system is able to create four-dimensional dynamically consistent with data sets or dynamic analyses (Rogers et al., 2013). The wind fields were nudged with the four-dimensional data assimilation (FDDA) using WMO surface meteorological observations and regional radiosondes in both March and October using methods described in Deng et al. (2009), but in March the wind fields were additionally nudged with High-Resolution Doppler Lidar (HRDL) and aircraft horizontal wind fields (see Karion et al. (2015) and Lauvaux et al. (2013) for additional details). Three nested domains were run at 1, 3, and 9 km resolution, with 50 vertical levels, and initial and boundary conditions from NOAA's Rapid Refresh (RAP) model. Fifty vertical terrain-following layers are used, with the center point of the lowest model layer located ~12 m above ground level (agl). The thickness of the layers increases gradually with height, with 27 layers below 850 hPa (~1550 m agl).

S2.2 WRF-Chem forward simulations

WRF-Chem simulations were performed using the passive tracer module described in previous studies (Díaz Isaac et al., 2014; Lauvaux et al., 2012), originally developed from the passive tracer option in WRF-Chem. The emissions of CH_4 from the Z-A inventory, assumed constant over time, are emitted at every model time step, and transported according to the mean horizontal and vertical winds, and turbulence fields. For the 9-km grid, the WRF-FDDA simulation (S2.1) was nudged to re-analysis fields and meteorological data from WMO surface stations and rawinsondes (i.e. wind speed and direction, and temperature above the PBL only) as well as airborne wind data, and Lidar wind profiles. For the WRF-Chem simulation, the 3-km grid was run with WRF but without FDDA to conserve mass within the domain. The simulation was re-initialized every 18 hours in both WRF-FDDA and WRF-Chem runs. The 3-dimensional fields of CH_4 mixing ratios were saved every hour to provide a continuous simulation over the time period (from 18:00 UTC on October 18, 2013 to 18:00 UTC on October 29, 2013). This time period was chosen because it encompassed the flights that showed the largest variability (in terms of the CH_4 enhancement) in the Lagrangian footprint-based simulations.

S2.3 HYSPLIT and STILT backward simulations

HYSPLIT and STILT are Lagrangian particle dispersion models that can be run off-line using archived transport fields from a meteorological model. HYSPLIT is often used for forward dispersion modeling, i.e. for modeling downwind concentration fields of pollutants from a known release point (Stein et al., 2015; Draxler and Hess, 1997). STILT (Lin et al., 2003) was developed based on HYSPLIT, but uses a different parametrization for vertical mixing to those choices available in HYSPLIT. It is commonly used for trace gas flux estimation using atmospheric inverse methods, because it was developed to run backwards in time to generate influence functions, or footprints. These can be used as the adjoint in an inverse model (Miller et al., 2013; Mueller et al., 2008). For the backwards runs, the HYSPLIT model was used with the setting to emulate the STILT model, that is to save the particle trajectories and variables required to produce "footprints", or influence functions, for each receptor, with units (ppm (μmol m^{-2} s^{-1})$^{-1}$). Integrated footprints from WRF-HYSPLIT for all the flight days are shown in Fig. S4.

We note that in both HYSPLIT and STILT the parameter that governs the minimum planetary boundary layer (PBL) depth (KMIX0) was set to 25 m, from the default 250 m. The STILT model was used with its default parameter list unless otherwise noted, but we note that the WRF-FDDA wind fields were not time-averaged, as is usually the case with coupled WRF-STILT model runs (Nehrkorn et al., 2010). Both models were run from receptors located every 30 seconds (approximately 2.1 km during level flight) along the flight tracks, i.e. following the aircraft's latitude, longitude, altitude, and time. At each receptor (particle origin), 2000 particles were released, and their trajectories followed back in time for 24 hours. After 24 hours, for all flights, the particles had little to no surface influence (i.e. had exited the boundary layer) in the study domain (the 25-county Barnett domain over which the inventory was developed, Fig. 1). Three combinations of these models were run for all the flights (Table 1): WRF-FDDA/HYSPLIT (also referred to as WRF-HYSPLIT), WRF-FDDA/STILT (or WRF-STILT), and NAM-HYSPLIT.

For two days, 20131019 and 20131028, additional configurations of WRF-FDDA/HYSPLIT and WRF-FDDA/STILT were tested. In HYSPLIT, the changing of the boundary layer turbulence parameterization (using KBLT) was investigated in both the forward and backward runs. The default in HYSPLIT is to use the Kantha-Clayson parametrization, but the use of the WRF turbulent kinetic energy (TKE) to determine the vertical mixing was also tested. We note that the TKE parametrization was corrected for an error in HYSPLIT 4 revision 931, so here we are using this latest update (dated 2018-02-02). These options are described in detail in Draxler and Hess (1997) and the HYSPLIT User Guide (https://www.arl.noaa.gov/documents/reports/HYSPLIT_user_guide.pdf). This vertical turbulence computational method was found to have little effect on results (Fig. S5), so we chose the default HYSPLIT configuration (Kantha-Clayson parametrization) for further analyses.

Other HYSPLIT parameters were set to the default values, including the use of heat and momentum fluxes from the driving model to determine boundary layer stability (KBLS=1). We found that changing the PBL depth determination from the default (using the PBL in the WRF-FDDA model, KMIXD=0) to a TKE-based PBL depth (KMIXD=2), did not make a difference in either flight. In the STILT model, changing the default PBL determination method from the default of using the Richardson number (KMIXD=3; note, this Richardson number method is not available in the

current HYSPLIT model) to using the PBL from the input WRF model (KMIXD=0), was also investigated but we found little difference between the two (not shown). Additionally, for 20131028 the effect of gridding footprints at a higher resolution of 0.04 degrees (and convolving with a higher-resolution 4 km inventory) vs. our default of 0.1 degrees was investigated. Lastly, we tested one configuration on 20131028 for which we released 5000 particles instead of 2000 at each receptor point, to see if the results were sensitive to this number. Neither the spatial resolution or particle number affected the predicted enhancements and are not discussed further here.

The original WRF-FDDA runs (conducted in 2013 for the initial campaign) were not configured specifically for running with the STILT model, which requires specific variable outputs and time-averaged wind fields. We were able to re-run WRF for the middle 3-km domain using the driver data from the 9-km domain in the original runs, but now outputting the proper variables for WRF-STILT coupling, for the last four flights: 20131019, 20131020, 20131025, and 20131028. For previous flights the driver data was unavailable from the original runs. For all eight flights, the STILT model was run with the original archived WRF-FDDA fields by bypassing the specific requirements for the WRF-STILT coupling and allowing STILT to use the fields that were provided, as it does when it is driven by non-WRF products, such as NAM. Using the averaged wind fields and other STILT-specific variables caused the footprint strength and enhancement to increase by 1% (20131019) to 40% (20131028), ranging between 0.2 ppb and 5 ppb in the average downwind enhancement for those four flights where both versions were compared (Fig. S5). Results shown for STILT in Fig. 2 and 3 are using the averaged fields; Fig. 4 shows the results from the averaged fields for the last four flights and instantaneous wind fields for the first four.

S2.4 WRF-HYSPLIT forward simulations

HYSPLIT was run using the above-described WRF-Chem simulations (S2.2) for their meteorological fields only (no tracer information), in forward mode. Unlike in the backward/footprint mode, here the particles were released from the emissions location, in this case at the center of each 0.1-degree cell from the Z-A inventory. The particles represent CH_4 emissions and were released with mass corresponding to the emission rate in each cell, in kg h^{-1}. We conducted two of these simulations; one beginning at 1:00 UTC 20131019 and ending at 4:00 UTC 20131023, and one beginning at 21:00 UTC 20131024 and ending at 23:00 UTC 20131028. The mole fraction fields were output hourly at 0.1-degree horizontal resolution and at 15 vertical levels (top of the lowest level was 50 m, and then from 100 m to 1000 m every 100 m, then 1200, 2000, 3000, and 10,000 m). We note that although the mole fractions were saved at these resolutions, the particle motion was resolved at the native WRF-Chem resolution (3 km, 50 levels, hourly, as described above) regardless of the choice of output resolution. We conducted the forward HYSPLIT simulations with the default Kantha-Clayson turbulent mixing parametrization. We also tested the turbulent kinetic energy-based (TKE) turbulence parametrization, but, similarly to the footprint/backward runs (Fig. S5), we did not see a large difference. We first confirmed that the CH_4 enhancements from the forward HYSPLIT model runs correspond to the backward runs at the observation locations (Fig. S6), and then used this forward model to compare with the four-dimensional WRF-Chem fields to more fully investigate model differences.

S2.5 WRF-LPDM backward simulations

The Lagrangian Particle Dispersion Model (LPDM) (Uliasz, 1994) was used to generate footprints from the WRF-FDDA transport fields. The turbulent motion in the Planetary Boundary Layer is parameterized following a Mellor-Yamada turbulence closure scheme to calculate the Lagrangian time scale and hence the vertical motion of particles near the surface. The turbulent motion is distributed in the horizontal and vertical directions assuming isotropic mixing in the horizontal plane. The energy dissipation rate is directly derived from the Turbulent Kinetic Energy values from WRF-FDDA and combined with the wind velocity variance to calculate the Lagrangian time scale. The LPDM (coupled with WRF) has been evaluated in previous studies by comparing WRF-Chem direct simulations of trace gases (e.g. Lauvaux et al., 2012) for daytime tower-based measurement locations, with significant discrepancies between the two models under stable and neutral conditions. Daytime differences under convective stability conditions were found to be low at weekly time scales (less than 5% of the observed model-data mismatches) but significant in terms of additional random errors at the hourly time scale (up to 50% of the observed model-data mismatches) (Lauvaux et al., 2012).

S2.6 WRF2-FLEXPART backward simulations

A second set of WRF model runs were performed to couple with FLEXPART ("FLEXible PARTicle dispersion model", https://www.flexpart.eu), another commonly-used Lagrangian particle trajectory model (Angevine et al., 2014; Brioude et al., 2013); we designate these as WRF2-FLEXPART (FP) in Table 1. For this set of runs, four different WRF configurations were run, testing two different PBL schemes and two different boundary / initial conditions. The four configurations are shown as GM, EM, GT, and ET in table 1 of Angevine et al. (2014). In brief, WRF version 3.5 was run on a single grid of 12-km horizontal spacing, with 60 vertical levels. All configurations used the RRTMG longwave and shortwave radiation schemes and Grell 3D cumulus scheme. The four configurations differed by the use of the MYNN (GM and EM) or TEMF (GT and ET) PBL schemes, and by the initialization data set. GM and GT were initialized with GFS analysis, while EM and ET were initialized with ERA-Interim. In Angevine et al. (2014) these configurations were shown to be statistically indistinguishable and can be treated as members of a (small) ensemble.

FLEXPART was run using each of these four WRF configurations as a driver, to generate footprints along the flight tracks that were convolved with the Z-A inventory to produce simulated CH_4 enhancements. The model released 3000 particles every 30 seconds along the flight tracks (as for the other Lagrangian footprint models), backwards in time, following them for 18 hours. The results shown in Fig. 2 and Fig. 3 of the main text are from the MYNN/ERA-Interim WRF run. The results shown in Fig. 4 of the main text for WRF2-FP are the average enhancement over the four different runs, with the error bar indicating their standard deviation. On 20131016, one of the four configurations (EM) gave very small (a factor of 2 or more lower) enhancements than the other three because of a too-shallow PBL in the model that predicted the aircraft was flying above the PBL for large portions of the downwind transects. For this day, this model run was not included in the averages.

S2.7 WRF-STILT CarbonTracker Lagrange (CT-L) backward simulations

An additional WRF/STILT coupled configuration, WRF-CTL/STILT (also referred to as CT-L in the figure captions and Table 1) was also tested for all but the first flight. WRF v3.6.1 runs were 10-km resolution, continental scale outputs conducted by Atmospheric and Environmental Research (AER) with configuration similar to Nerkhorn et al. (2010) for NOAA's Carbon-Tracker Lagrange (CT-L; https://www.esrl.noaa.gov/gmd/ccgg/carbontracker-lagrange/) project. Analysis data from the NOAA NCEP North American Regional Reanalysis (NARR), 32 km grids was used to provide initial and boundary conditions for the WRF runs. Forecasts were reinitialized every 24 h, and analysis nudging to NARR every 3 h was used to constrain the model solution. WRF used the Noah land surface model and the Yongsei University (YSU) PBL scheme, with the RRTMG longwave and shortwave radiation and Grell-Devenyi convection option. STILT footprints were generated by AER for these flights every 60 s along the track, with 500 particles per receptor, and the near-field footprints were used for this analysis, which were output hourly for 24 h back at 0.1-degree resolution.

S3 Bayesian inversion: construction of error covariance matrices R and B

First, a series of inversions was completed for different combinations of the variances along the diagonals of the **R** and **B** matrices (Eq. (4)) to determine the sensitivity of the inversion results to these parameters (Fig. S7). The variances in **R** were constant throughout each flight (i.e. constant along the diagonal), while the diagonal terms of **B** were considered as a multiple of the 1-sigma uncertainty on the Z-A inventory (the 95% confidence interval as provided by the authors, the 1-sigma uncertainty ranged from 5-35% of each grid cell's value), squared to obtain a variance for use in **B**. Off-diagonal elements (i.e. correlations) in either **R** or **B** were outside the scope of this work, and not considered.

We only model **R** and **B** as diagonal matrices here, with no correlation between observations, for simplicity. Significant work has been devoted to identifying the proper covariance structure for these correlation matrices (Wu et al., 2013; Bousserez et al., 2015; Lauvaux et al., 2016), and we would expect correlations to be especially important in small spatial domains such as this one. However, given the range of posterior results and their sensitivity to our choice of **R** and **B**, we do not expect the overall trend of model-based emissions being higher than inventory estimates to change based on the structure of these covariance matrices in the inversion; likely these correlations would change the relative weighting of the result toward either the data or the prior within the range of solutions shown in Fig. S7.

After the sensitivity analysis, two other options were investigated for determining these matrices: Restricted Maximum Likelihood (RML) and the variance of the model ensemble. First, the **R** and **B** diagonal values were estimated using RML (Michalak et al., 2005); we used the method to simultaneously optimize for a single variance for **R** per flight and a single multiplier on the inventory uncertainty (on the standard deviation) for **B**. We placed an upper limit of 10 times the inventory uncertainty on each pixel, and the RML chose that upper limit on all flights. We did not allow the RML to choose a higher multiplier on the inventory uncertainty because we did not believe it would be realistic to consider that the authors' uncertainty estimates were more than a factor of 10 too low. Thus, **B** was the same for all flights, and generally a function of the emission rate in each grid cell (Fig. S8, lower right). RML-derived standard

deviations for **R** ranged from 11 to 70 ppb, all values above the uncertainty on CH_4 enhancements from the observations.

Given the wide range of CH_4 enhancements predicted by the various transport and dispersion models, we also conduct inversions choosing the model-data mismatch matrix (**R**) proportional to the spread in the enhancements from the various models. We used the variance of the modeled enhancements at each observation location and time, summed with the variance from the background uncertainty from Karion et al. (2015), to construct an **R** matrix with different values along the diagonal. We used the following five models to construct this variance: WRF-HYSPLIT, WRF-STILT, WRF-LPDM, CT-L, and NAM-HYSPLIT, as they were available for all eight flights, with the exception of 20130325 which did not have CT-L results, so the other four were used for this day. For this inversion, the matrix **B** was kept at 10 times the inventory uncertainty. Figure S7 shows the results of the full sensitivity test of **R** and **B** on the total posterior emissions. We chose to show the result of the RML-based parameters in Figs. 8 and 9 in the main text.

Figure S8 shows the flux corrections (posterior-prior) for the eight flight days from inversions with the RML-estimated **R** and **B**, along with the spatial map of the diagonal terms in **B**, the prior error covariance matrix. The spatial pattern of adjustments to the inventory follows the pattern of the prior error, as one would expect in this framework.

Supplementary Tables

Table S1. Comparison of WRF-FDDA wind speed and wind direction with winds measured by the aircraft (AC) and by ground-based High-Resolution Doppler Lidar (HRDL). Aircraft measurement comparisons are made by sampling the model at the aircraft location and time, only for locations within the model's PBL; errors shown are averaged over the entire flight. HRDL measurement comparisons are made by averaging both the HRDL and WRF winds through the model's PBL; errors shown are averaged for 12 h prior to the flight's start. Errors are the measured minus model variable, shown as a mean ± standard deviation of this difference. Days with significant wind speed differences are highlighted in gray.

Flight Number	AC-WRF wind speed error (m/s)	AC-WRF wind speed error (%)	AC-WRF wind direction error (°)	HRDL-WRF wind speed error (m/s)	HRDL-WRF wind speed error (%)	HRDL-WRF wind direction error (°)
20130325	-0.1 ± 1.3	-1.7 ± 17.4	5 ± 13	0.2 ± 1.1	3.4 ± 17.8	-14 ± 35
20130327	-0.9 ± 1.9	-7.6 ± 15.7	1 ± 7	-0.5 ± 1.2	-6.7 ± 14.6	-1 ± 7
20130330	-0.1 ± 1.9	-1.6 ± 31.0	-21 ± 40	0.3 ± 3.0	3.9 ± 45.6	-25 ± 53
20131016	-1.8 ± 1.7	-30.4 ± 29.5	-2 ± 9	NA	NA	NA
20131019	-1.1 ± 1.4	-22.3 ± 28.1	4 ± 12	NA	NA	NA
20131020	-0.1 ± 1.3	-0.5 ± 12.7	0 ± 6	NA	NA	NA
20131025	-1.0 ± 1.1	-20.4 ± 22.9	-4 ± 17	-0.1 ± 0.9	-1.2 ± 13.8	4 ± 8
20131028	-0.7 ± 1.4	-7.9 ± 15.3	6 ± 8	0.9 ± 0.9	16.5 ± 15.7	11 ± 13

Table S2. Differences between WRF-FDDA PBL depth and PBL depth determined from airborne observations (2nd and 3rd columns) and HRDL averages (4th and 5th columns) over 12 h, expressed both in meters and as a percent of the observed depth. Differences shown are the mean ± standard deviation of the difference for each flight.

Flight Number	AC-WRF PBL difference (m)	AC-WRF PBL difference (%)	HRDL-WRF PBL difference (m)	HRDL-WRF PBL difference (%)
20130325	-385 ± 179	-33 ± 15	121 ± 180	24 ± 35
20130327	558 ± 180	34 ± 11	105 ± 34	48 ± 15
20130330	280 ± 165	25 ± 14	398 ± 662	43 ± 72
20131016	-47 ± 123	-6 ± 17	NA	NA
20131019	312 ± 122	31 ± 12	NA	NA
20131020	211 ± 61	25 ± 7	NA	NA
20131025	-78 ± 44	-11 ± 6	-76 ± 207	-18 ± 49
20131028	147 ± 21	23 ± 3	143 ± 189	31 ± 41
Mean	125 ± 289	11 ± 14	138 ± 169	26 ± 26

Table S3. Coefficients of determination (R^2) for each of the tested models. For WRF-Chem the R^2 value sampling the model at an altitude 200 m lower than the flight path is shown in parentheses after the value for the model sampled at the flight altitude. Empty cells exist if a given model was not run for a given day.

Flight Number	WRF-HYSPLIT	WRF-STILT	WRF-LPDM	NAM-HYSPLIT	CT-L	WRF-Chem	WRF2-FP (MYNN/ERA-I)
20130325	0.09	0.09	0.07	0.03			
20130327	0.18	0.06	0.19	0.13	0.07		
20130330	0.05	0.05	0.00	0.09	0.24		
20131016	0.29	0.14	0.08	0.43	0.00		0.02
20131019	0.26	0.03	0.30	0.56	0.57	0.13 (0.49)	0.39
20131020	0.04	0.09	0.39	0.04	0.36	0.13	0.01
20131025	0.35	0.41	0.35	0.09	0.44	0.56	0.10
20131028	0.47	0.16	0.48	0.55	0.58	0.26 (0.37)	0.51

Supplementary Figures

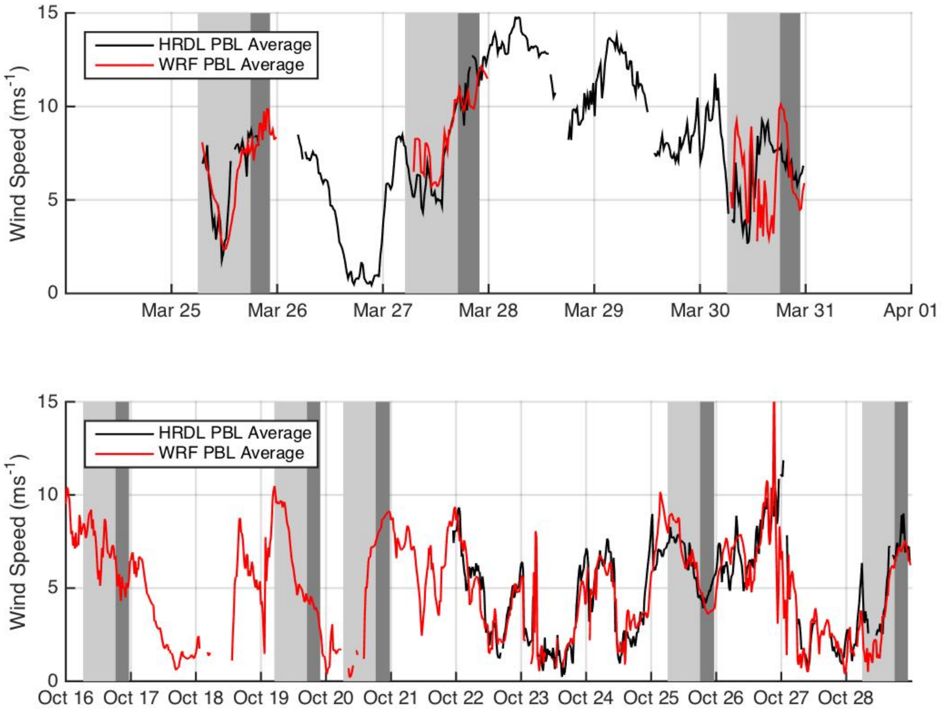

Figure S1. Average PBL wind speed time series from WRF-FDDA (red) and HRDL (black). Dark gray shading indicates flight periods; light gray indicates the 12 h period prior to the flight start that was used in the comparisons with HRDL, shown in Table 1. HRDL was not operating from Oct. 16 to Oct. 22.

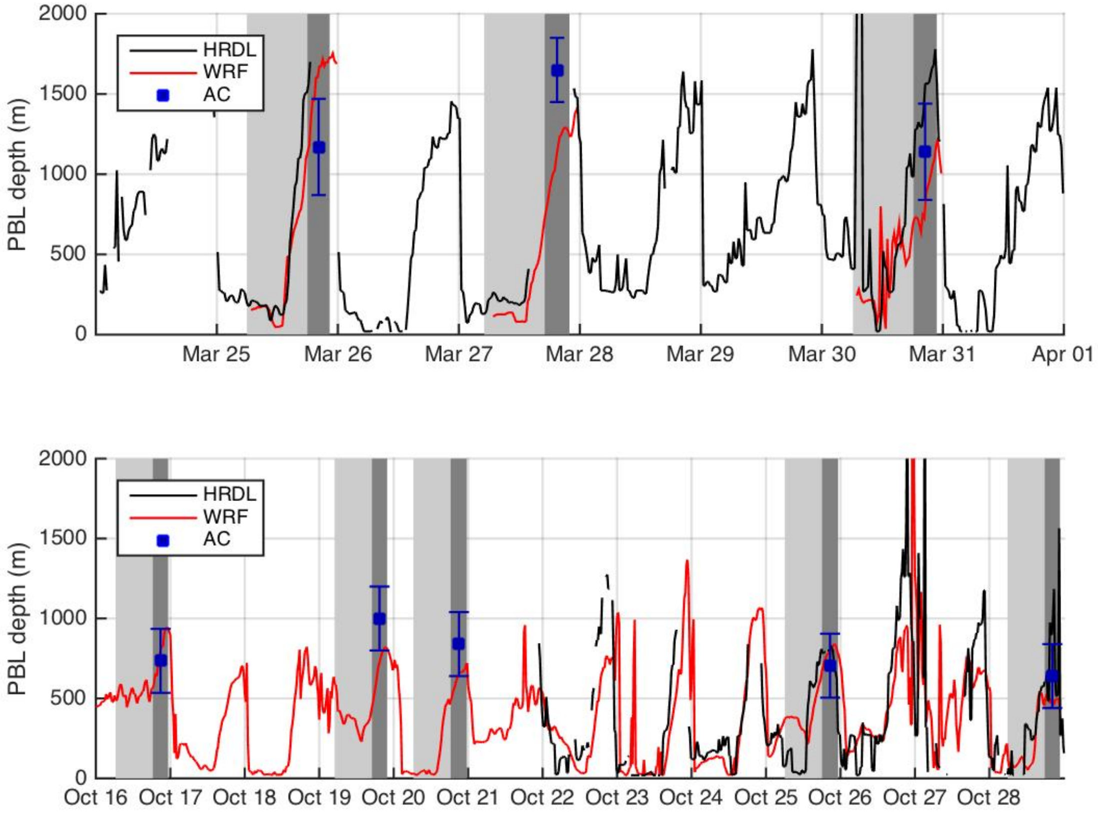

Figure S2. Average PBL depth time series from WRF-FDDA (red) and HRDL (black). Dark blue points and error bars indicate the PBL depth and variability used for the MB estimate, based on vertical profiles conducted by the aircraft. Dark gray shading indicates flight periods; light gray indicates the 12 h period prior to the flight (times are in UTC, or local time + 5 h).

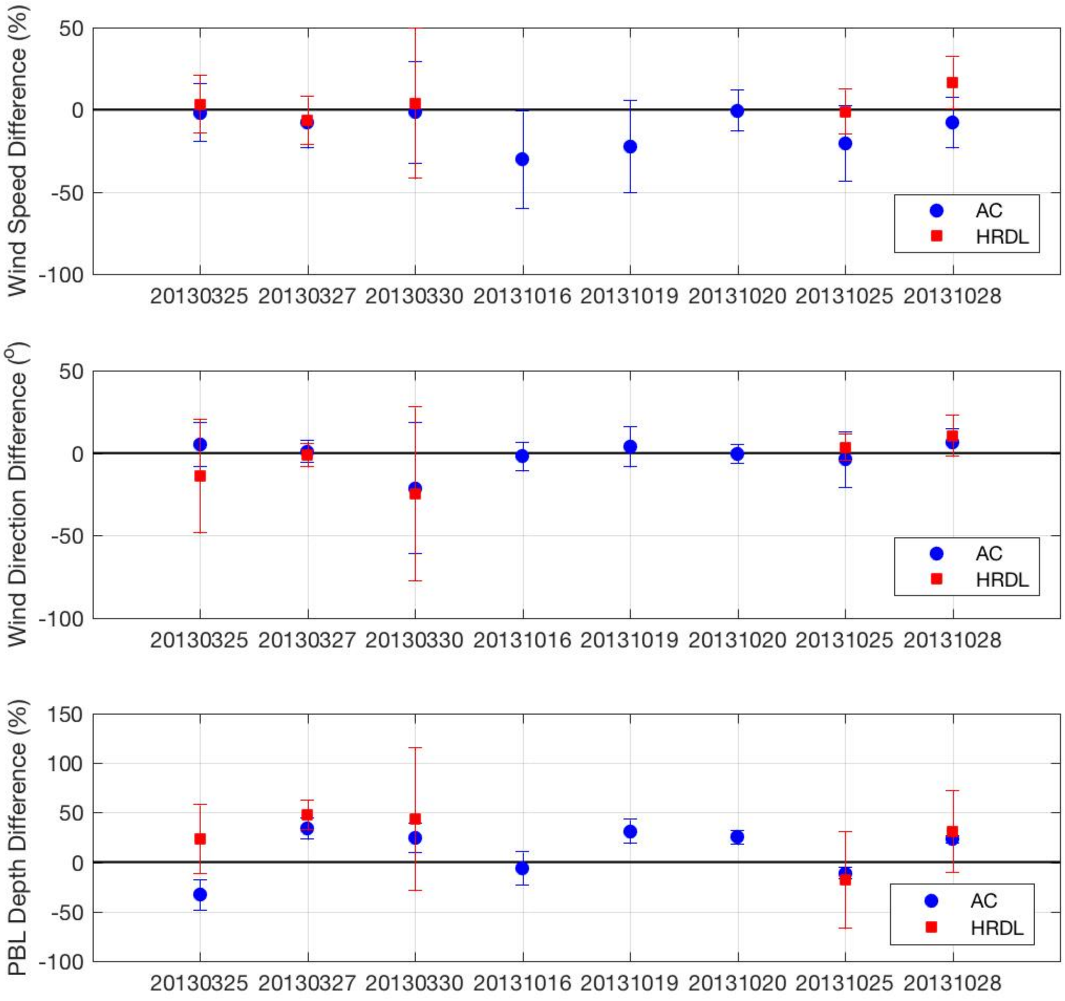

Figure S3. Differences between WRF-FDDA wind speed (top), wind direction (middle), and PBL depth (bottom) and airborne (blue) or HRDL (red) observations. In all plots, the model values are subtracted from the observations. Error bars are one standard deviation of the variability around the average differences. HRDL comparisons are made for 12 h prior and up to the end of the flight; aircraft comparisons are averaged over the duration of the flight.

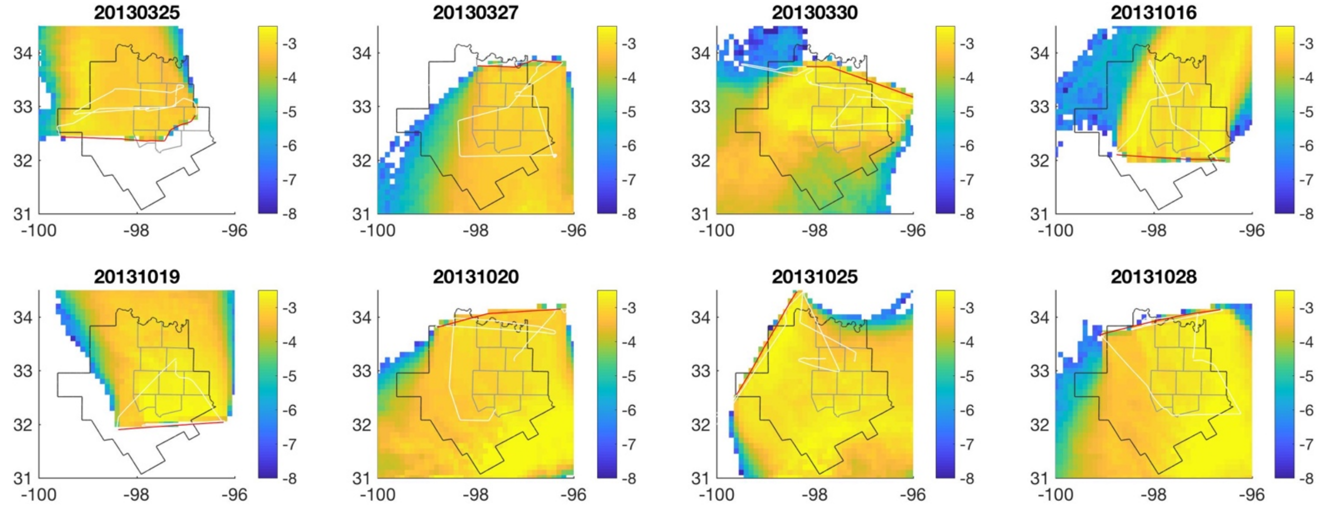

Figure S4. Averaged footprints (log scale) for the downwind transects for each flight, from WRF-FDDA/HYSPLIT model. The 25-county Barnett region is outlined in gray, with the 8 core counties in light gray. Downwind flight transects are shown by red lines, with the entire flight track in white. The footprint colorbar units are in log (ppb (nmol $(m^2s)^{-1})^{-1}$).

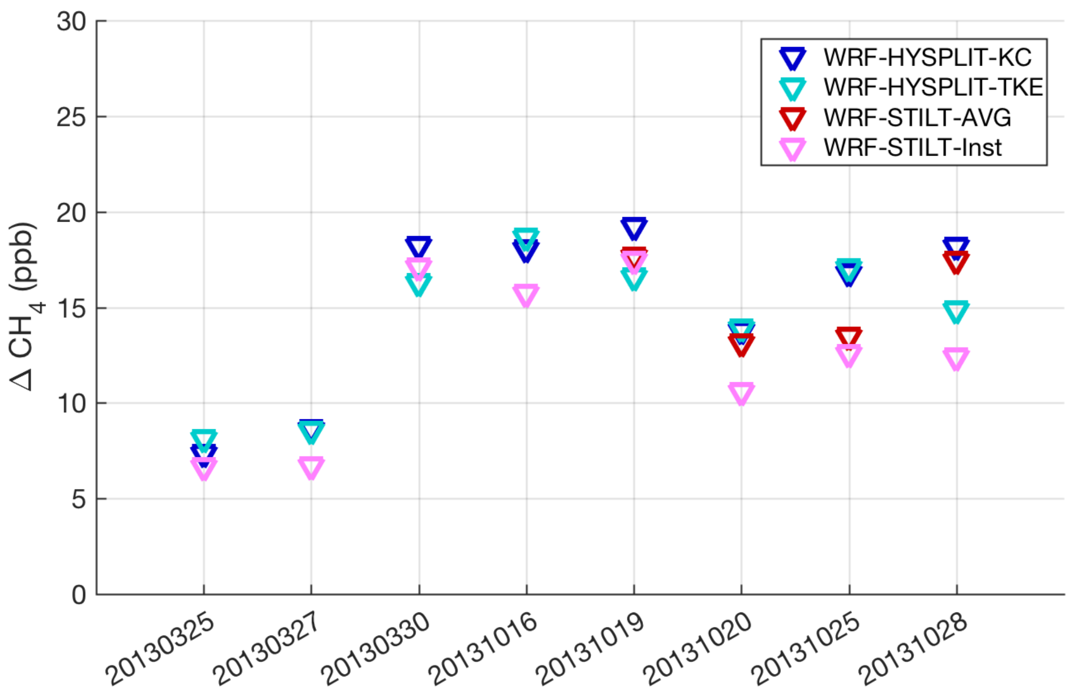

Figure S5. Average downwind CH_4 enhancement from two different boundary layer turbulence parametrizations in HYSPLIT: default Kantha-Clayson (WRF-HYSPLIT-KC, dark blue), and TKE-based (WRF-HYSPLIT-TKE, light blue). Average downwind CH_4 enhancements from two version for WRF-STILT are also shown: using averaged winds for the four flights that they were available (red, 20131019, 20, 25, and 28), and using instantaneous winds at 20-minute intervals (pink).

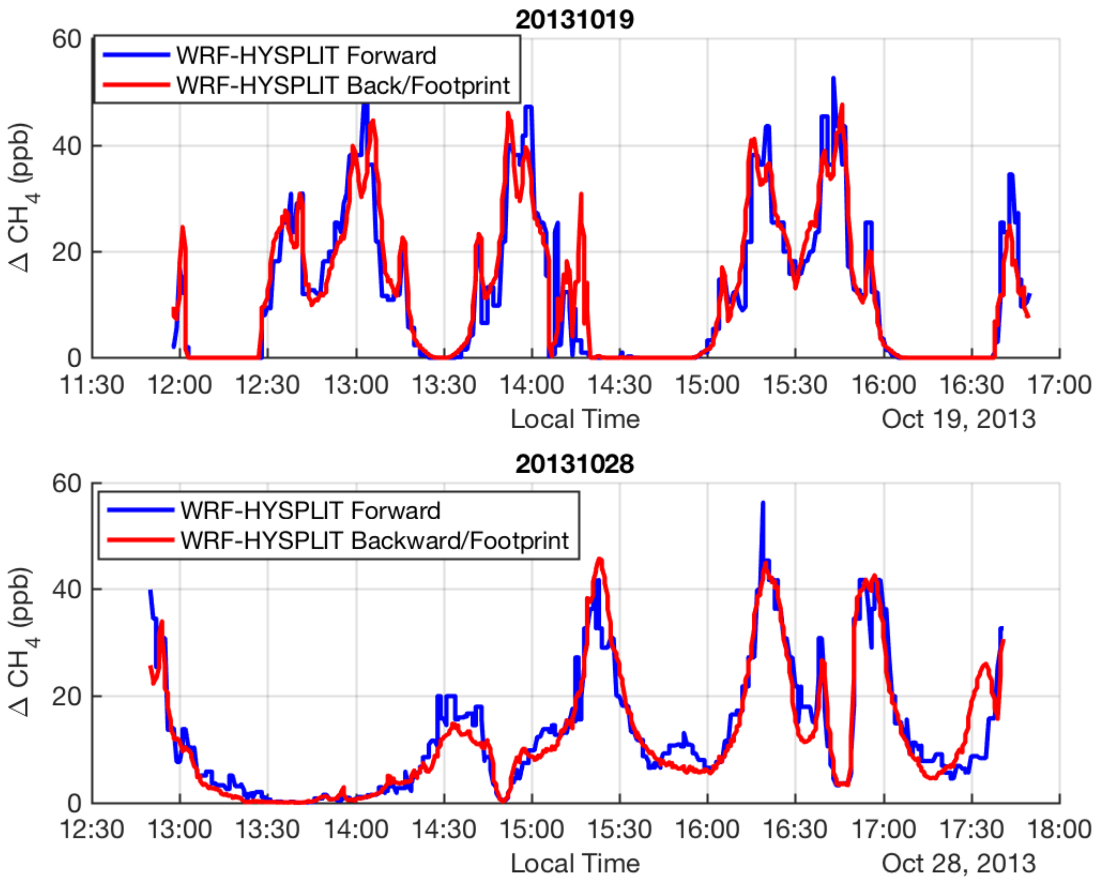

Figure S6. Comparison of WRF-HYSPLIT forward and backward simulations time series along aircraft flight path on 20131019 (top) and 20131028 (bottom). The forward run is driven by the WRFChem 3-km wind fields, while the backward run is driven by the nested 1-km and 3-km original WRF-FDDA fields. Forward run mole fraction fields output at 0.1-degree and hourly resolution were interpolated onto the location of the aircraft, leading to a less smooth appearance. The similarity between the two runs indicates that the forward and backward simulations of HYSPLIT are indeed equivalent, but also that the resolution of the WRF model driving them is not affecting the results.

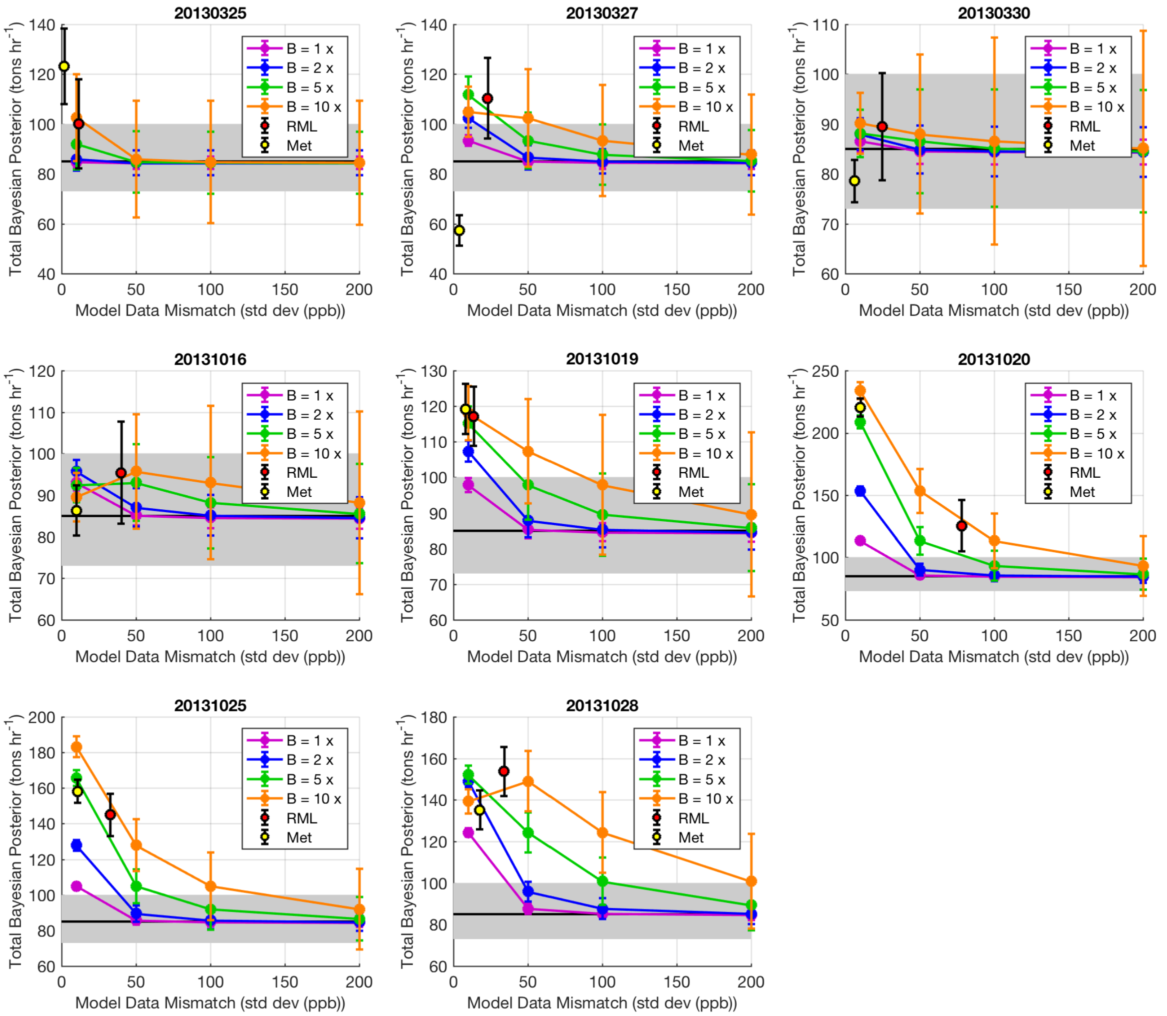

Figure S7. Sensitivity of Bayesian inversion posterior (total emissions summed over domain) for each flight to the model-data mismatch (R) standard deviation for various values of the prior error covariance, B, as a multiplier of the inventory uncertainty. B is assumed to be diagonal and a product of a constant and the inventory uncertainty, ranging from one to 10 times the 1-sigma inventory uncertainty (different for each pixel in the domain). R is also assumed to be diagonal, and constant for each flight, with the standard deviation (square root of variance along the diagonal) ranging from 10 ppb to 200 ppb CH_4. The posterior total value for R determined from the RML optimization with B=10x is also indicated on the figure (black and red circle). The posterior total value for R determined using the variance of the meteorological models with B=10x is shown for the mean value of the standard deviation along the diagonal (black and yellow circle). Note the y-axis is scaled differently for each flight. The prior total is equal to the inventory (85 t h^{-1}) (black line), shown along with its 95% confidence intervals (gray shading). Error bars on the total emissions are also 95% confidence intervals, from the posterior uncertainty (Eq. 5).

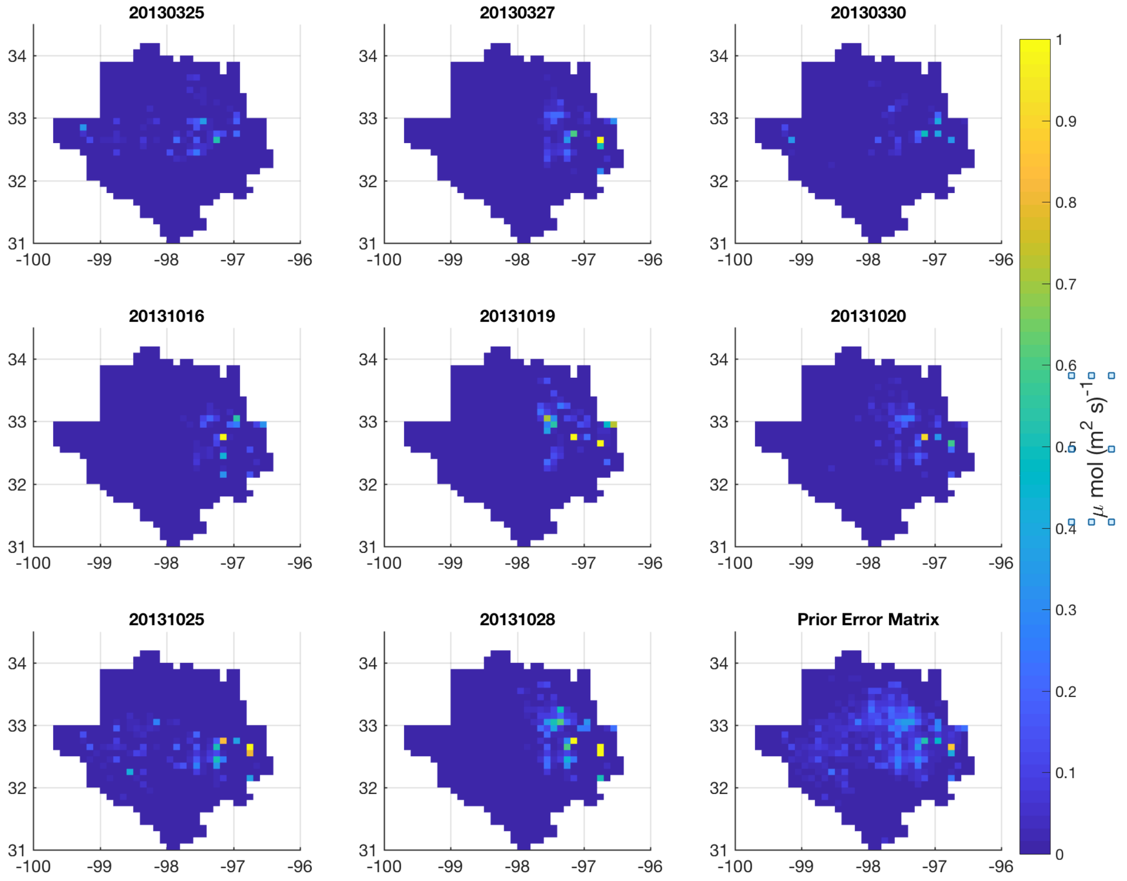

Figure S8. Map of flux corrections to the prior (i.e. posterior – inventory), for each day, as labeled, from inversion using RML-estimated parameters. Lower right corner figure shows the prior error covariance matrix B, at 10 times the 1-sigma inventory uncertainty. Note: The color scale has been truncated for clarity.

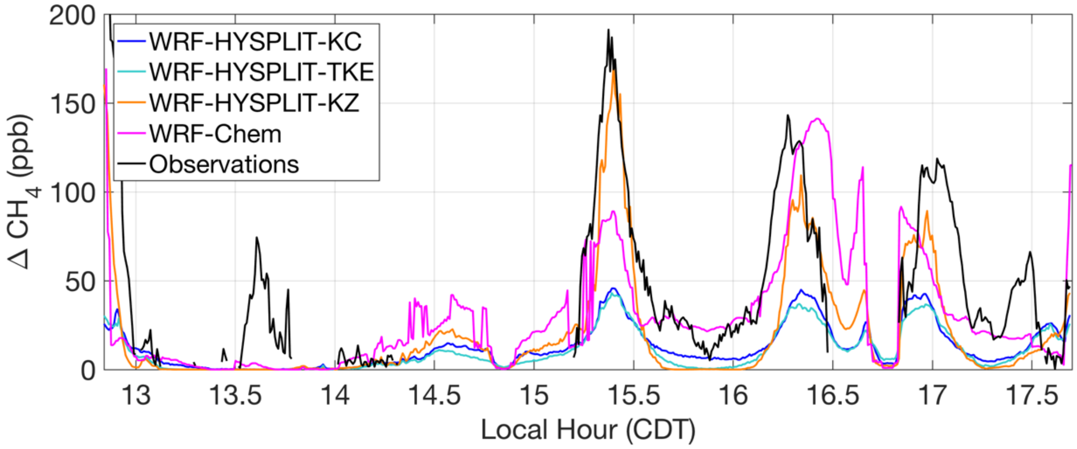

355 **Figure S9. Modeled CH_4 enhancements along the flight track on 20131028, from three different HYSPLIT parametrizations for turbulent mixing in the PBL: Kantha-Clayson (KC, dark blue), TKE (light blue), and a new experimental parametrization utilizing the vertical eddy diffusivity from WRF (KZ, orange). WRF-Chem (pink) and observations (black) are also shown for comparison, as in Fig. 3 in the main text.**

360

Supplementary Reference List

Angevine, W. M., Brioude, J., McKeen, S., and Holloway, J. S.: Uncertainty in Lagrangian pollutant transport simulations due to meteorological uncertainty from a mesoscale WRF ensemble, Geosci. Model Dev., 7, 2817-2829, 10.5194/gmd-7-2817-2014, 2014.

Bonin, T. A., Choukulkar, A., Brewer, W. A., Sandberg, S. P., Weickmann, A. M., Pichugina, Y. L., Banta, R. M., Oncley, S. P., and Wolfe, D. E.: Evaluation of turbulence measurement techniques from a single Doppler lidar, Atmos. Meas. Tech., 10, 3021-3039, 10.5194/amt-10-3021-2017, 2017.

Bousserez, N., Henze, D. K., Perkins, A., Bowman, K. W., Lee, M., Liu, J., Deng, F., and Jones, D. B. A.: Improved analysis-error covariance matrix for high-dimensional variational inversions: application to source estimation using a 3D atmospheric transport model, Quarterly Journal of the Royal Meteorological Society, 141, 1906-1921, 10.1002/qj.2495, 2015.

Brioude, J., Arnold, D., Stohl, A., Cassiani, M., Morton, D., Seibert, P., Angevine, W., Evan, S., Dingwell, A., Fast, J. D., Easter, R. C., Pisso, I., Burkhart, J., and Wotawa, G.: The Lagrangian particle dispersion model FLEXPART-WRF version 3.1, Geosci. Model Dev., 6, 1889-1904, 10.5194/gmd-6-1889-2013, 2013.

Deng, A., Stauffer, D., Gaudet, B., Dudhia, J., Hacker, J., Bruyere, C., Wu, W., Vandenberghe, F., Liu, Y., and Bourgeois, A.: Update on WRF-ARW end-to-end multi-scale FDDA system, 10th Annual WRF Users' Workshop, Boulder, CO, 2009, 2009.

Díaz Isaac, L. I., Lauvaux, T., Davis, K. J., Miles, N. L., Richardson, S. J., Jacobson, A. R., and Andrews, A. E.: Model-data comparison of MCI field campaign atmospheric CO2 mole fractions, Journal of Geophysical Research: Atmospheres, 119, 10536-10551, 10.1002/2014JD021593, 2014.

Draxler, R. R., and Hess, G. D.: Description of the Hysplit_4 Modeling System, NOAA Air Resources Laboratory, Silver Spring, MD, NOAA Technical Memorandum, www.arl.noaa.gov/documents/reports/arl-224.pdf, , 1997.

Grund, C. J., Banta, R. M., George, J. L., Howell, J. N., Post, M. J., Richter, R. A., and Weickmann, A. M.: High-Resolution Doppler Lidar for Boundary Layer and Cloud Research, J. Atmos. Ocean. Technol., 18, 376-393, 10.1175/1520-0426(2001)018<0376:hrdlfb>2.0.co;2, 2001.

Karion, A., Sweeney, C., Kort, E. A., Shepson, P. B., Brewer, A., Cambaliza, M., Conley, S. A., Davis, K., Deng, A. J., Hardesty, M., Herndon, S. C., Lauvaux, T., Lavoie, T., Lyon, D., Newberger, T., Petron, G., Rella, C., Smith, M., Wolter, S., Yacovitch, T. I., and Tans, P.: Aircraft-Based Estimate of Total Methane Emissions from the Barnett Shale Region, Environ. Sci. Technol., 49, 8124-8131, 10.1021/acs.est.5b00217, 2015.

Lauvaux, T., Schuh, A. E., Uliasz, M., Richardson, S., Miles, N., Andrews, A. E., Sweeney, C., Diaz, L. I., Martins, D., Shepson, P. B., and Davis, K. J.: Constraining the CO2 budget of the corn belt: exploring uncertainties from the assumptions in a mesoscale inverse system, Atmos. Chem. Phys., 12, 337-354, 10.5194/acp-12-337-2012, 2012.

Lauvaux, T., Deng, A., Gaudet, B., Sweeney, C., Petron, G., Karion, A., Brewer, A., Hardesty, M., Herndon, S., and Yacovitch, T.: Quantification of methane sources in the Barnett Shale (Texas) using the Penn State WRF-Chem-FDDA realtime modeling system, 14th WRF User's Workshop, Boulder, CO, 2013, 2013.

Lauvaux, T., Miles, N. L., Deng, A. J., Richardson, S. J., Cambaliza, M. O., Davis, K. J., Gaudet, B., Gurney, K. R., Huang, J. H., O'Keefe, D., Song, Y., Karion, A., Oda, T., Patarasuk, R., Razlivanov, I., Sarmiento, D., Shepson, P., Sweeney, C., Turnbull, J., and Wu, K.: High-resolution atmospheric inversion of urban CO2 emissions during the dormant season of the Indianapolis Flux Experiment (INFLUX), J. Geophys. Res.-Atmos., 121, 5213-5236, 10.1002/2015jd024473, 2016.

Lin, J. C., Gerbig, C., Wofsy, S. C., Andrews, A. E., Daube, B. C., Davis, K. J., and Grainger, C. A.: A near-field tool for simulating the upstream influence of atmospheric observations: The Stochastic Time-Inverted Lagrangian Transport (STILT) model, Journal of Geophysical Research: Atmospheres, 108, n/a-n/a, 10.1029/2002JD003161, 2003.

Michalak, A. M., Hirsch, A., Bruhwiler, L., Gurney, K. R., Peters, W., and Tans, P. P.: Maximum likelihood estimation of covariance parameters for Bayesian atmospheric trace gas surface flux inversions, J. Geophys. Res.-Atmos., 110, 16, 10.1029/2005jd005970, 2005.

Miller, S. M., Wofsy, S. C., Michalak, A. M., Kort, E. A., Andrews, A. E., Biraud, S. C., Dlugokencky, E. J., Eluszkiewicz, J., Fischer, M. L., Janssens-Maenhout, G., Miller, B. R., Miller, J. B., Montzka, S. A., Nehrkorn, T., and Sweeney, C.: Anthropogenic emissions of methane in the United States, Proceedings of the National Academy of Sciences, 110, 20018-20022, 10.1073/pnas.1314392110, 2013.

Mueller, K. L., Gourdji, S. M., and Michalak, A. M.: Global monthly averaged CO2 fluxes recovered using a geostatistical inverse modeling approach: 1. Results using atmospheric measurements, J. Geophys. Res.-Atmos., 113, 15, 10.1029/2007jd009734, 2008.

Nehrkorn, T., Eluszkiewicz, J., Wofsy, S. C., Lin, J. C., Gerbig, C., Longo, M., and Freitas, S.: Coupled weather research and forecasting–stochastic time-inverted lagrangian transport (WRF–STILT) model, Meteorol. Atmos. Phys., 107, 51-64, 10.1007/s00703-010-0068-x, 2010.

Stein, A. F., Draxler, R. R., Rolph, G. D., Stunder, B. J. B., Cohen, M. D., and Ngan, F.: NOAA'S HYSPLIT Atmospheric Transport and Dispersion Modeling System, Bull. Amer. Meteorol. Soc., 96, 2059-2077, 10.1175/bams-d-14-00110.1, 2015.

Uliasz, M.: Lagrangian particle modeling in mesoscale applications, in: Environmental Modeling II, edited by: Zanetti, P., Computational Mechanics Publications, 71-102, 1994.

Wu, L., Bocquet, M., Chevallier, F., Lauvaux, T., and Davis, K.: Hyperparameter estimation for uncertainty quantification in mesoscale carbon dioxide inversions, Tellus B: Chemical and Physical Meteorology, 65, 20894, 10.3402/tellusb.v65i0.20894, 2013.
